# Insomnia Among Adolescents in Northern Peru: Associations with Psychosocial, Health-Related, and Educational Factors in a Cross-Sectional Study Across Five Schools

**DOI:** 10.3390/jcm15041505

**Published:** 2026-02-14

**Authors:** Mario J. Valladares-Garrido, Palmer J. Hernández-Yépez, Angie Giselle Morocho Alburqueque, Luz A. Aguilar-Manay, Jassmin Santin Vásquez, Renzo Acosta-Porzoliz, Danai Valladares-Garrido, Darwin A. León-Figueroa, César J. Pereira-Victorio, Miguel Villegas-Chiroque, Víctor J. Vera-Ponce, Oriana Rivera-Lozada, Jean Pierre Zila-Velasque

**Affiliations:** 1 Escuela de Medicina Humana, Universidad Señor de Sipán, Chiclayo 14001, Peru; mvillegasch@uss.edu.pe (M.V.-C.); riveraoriana@uss.edu.pe (O.R.-L.); 2 Departamento de Ciencias Médicas, Facultad de Ciencias de la Salud, Universidad de Castilla-La Mancha, 13071 Ciudad Real, Spain; 3 Facultad de Medicina, Universidad Privada Norbert Wiener, Lima 15025, Peru; palmerxvii@gmail.com; 4 Facultad de Ciencias de la Salud, Universidad Nacional de Piura, Piura 20001, Peru; giselle.m.a.020@gmail.com; 5 Facultad de Medicina, Universidad San Martin de Porres, Chiclayo 14001, Peru; luz_aguilar2@usmp.pe (L.A.A.-M.); jassmin_santin@usmp.pe (J.S.V.); dalefi19@gmail.com (D.A.L.-F.); 6 EpiHealth Research Center for Epidemiology and Public Health, Lima 15001, Peru; renzo.acosta@upch.pe (R.A.-P.); danai.paola@gmail.com (D.V.-G.); 7 Facultad de Medicina, Universidad Peruana Cayetano Heredia, Lima 15313, Peru; 8 Escuela de Medicina, Universidad Cesar Vallejo, Trujillo 13011, Peru; 9 Facultad de Medicina, Universidad Continental, Lima 15046, Peru; pereira.victorio.cj@gmail.com; 10 Facultad de Medicina (FAMED), Universidad Nacional Toribio Rodríguez de Mendoza de Amazonas, Chachapoyas 01001, Peru; victor.vera@untrm.edu.pe; 11 Red Latinoamericana de Medicina en la Altitud e Investigación (REDLAMAI), Pasco 19001, Peru; zilavelasqueje@gmail.com; 12 Facultad de Medicina Humana, Universidad Nacional Daniel Alcides Carrion, Pasco 19001, Peru

**Keywords:** insomnia, sleep quality, mental health, adolescents, COVID-19, pandemic

## Abstract

**Background/Objectives:** Insomnia is common among adolescents and is associated with emotional, behavioral, and academic difficulties. Although high rates have been reported globally, evidence in Latin America—particularly in Peru—remains limited and heterogeneous. Many previous studies relied on small samples, descriptive designs, omitted key psychosocial variables, or were conducted during early pandemic waves, despite the rise in sleep disturbances following COVID-19 restrictions. This study aimed to estimate the prevalence of insomnia and identify associated factors among adolescents in northern Peru. **Methods:** An analytical cross-sectional study was conducted using secondary data from students attending five schools in Lambayeque, Peru. Insomnia was assessed using the Insomnia Severity Index (ISI). Sociodemographic, psychosocial, behavioral, and health-related variables—including self-esteem, family dysfunction, eating disorders, acne severity, mental health help-seeking, and digital behavior—were evaluated. Generalized linear models estimated prevalence ratios (PRs) and 95% confidence intervals (CIs). **Results:** Among 1313 adolescents (54.3% male; mean age 14.6 years), the prevalence of insomnia was 38.9% (95% CI: 36.1–41.5). In adjusted analyses, insomnia was associated with urban residence, non-Catholic religion, seeking mental health support, high social media use, internet use of 6–10 h/day, low self-esteem, eating disorders, greater acne severity, and experiencing the death of a family member due to COVID-19. **Conclusions:** Nearly four in ten adolescents reported insomnia, influenced by sociodemographic, psychosocial, and lifestyle-related factors. These findings provide updated post-pandemic evidence for the Peruvian context and highlight the multifactorial nature of adolescent insomnia. Further research is needed to clarify causal pathways and understand the long-term mental health implications of large-scale stressors such as the COVID-19 pandemic.

## 1. Introduction

In recent years, sleep disorders have gained great relevance because they impact daily life, health and medical care [1]. Insomnia is one of these disorders, which consists of having difficulties falling asleep, staying asleep or achieving good quality sleep in the general population [2]. In adolescents, insomnia is a common problem, as studies have found that its prevalence ranges between 9.6% and 17.0% [3]. In turn, it has been seen that the prevalence rates of insomnia symptoms are high and range from 40.0% to 66.0% in adolescents, while the prevalence rates for the complete diagnosis of this disorder vary from 9.0% to 23.0% [4]. Previous studies have reported varying prevalence rates of insomnia among adolescents, including Portugal (13.4%) [5], Norway (23.8%) [3], Spain (43.1%) [6], China (26.0%) [7], and Canada (38.8%) [8]. In Latin America, insomnia prevalence has been reported in Chile (66.7%) [9], Ecuador (42.0%) [10], Colombia (16.0%) [11], Brazil (22.0%) [12], and Mexico (35.2%) [13]. Within Peru, studies conducted in Trujillo (30.0%) [14], Chiclayo (4.4%) [15], Lima (12.0%) [16], and Huancayo (58.2%) [17] have also documented substantial variability in insomnia prevalence among adolescents.

There are different factors that can generate or increase the risk of having insomnia in adolescents [8]. Among these are unhealthy eating [6], feelings of anguish [8], domestic violence [14], immoderate consumption of coffee and alcohol [5], lack of communication and parental supervision [18] and fragmented or disturbed sleep with behavioral problems [19]. In turn, age (≥16 years) [5] and the female sex [5] have been associated with a higher prevalence of insomnia [20]. Additionally, factors associated with a lower prevalence of insomnia have been described, such as freedom to organize one’s own schedules [21], good family organization [21], fewer technological distractions [11], good mood [11] and greater family support [11].

However, there is still inconclusive evidence on the prevalence of insomnia and its associated factors in adolescents, even more so in a potential post-COVID-19 pandemic scenario that has increased the rates of this disorder by 18.5% [8] due to mandatory quarantines and closures of schools and recreation centers in the early years of the pandemic. While previous studies have documented the question of interest, they present several limitations [16]. First, they have a small sample size [22,23,24], and even have a descriptive approach [11,25]. Additionally, previous studies have information bias since they have not measured variables potentially associated with insomnia in adolescents—religion [3], self-esteem [3], internet use [18] and family dysfunction [16]—all of which are investigated in our research, or prior studies have used non-validated instruments [5]. Finally, few studies have evaluated the presence of this disorder three years after the COVID-19 pandemic, given that the vast majority have been addressed in the first pandemic waves [8,15].

Although previous studies have documented an increase in insomnia among adolescents during the COVID-19 pandemic, important gaps remain in the current literature. Many existing studies were conducted during the early phases of the pandemic, focused on limited sets of variables, or examined isolated risk factors without integrating psychosocial, behavioral, health-related, and contextual dimensions. Given that adolescence represents a critical developmental stage in which sleep patterns, emotional regulation, and health-related behaviors are consolidated, a comprehensive and updated examination of insomnia and its associated factors is warranted.

Moreover, evidence from Latin American settings remains scarce, despite marked social inequalities, differential access to digital technologies, and pandemic-related stressors that may uniquely shape adolescent sleep health. Understanding how psychosocial factors (such as self-esteem, family functioning, and resilience), behavioral patterns (including internet and social media use), and health-related conditions interact in relation to insomnia is essential to inform targeted prevention and mental health promotion strategies. In this context, the present study aims to provide an integrative analysis of factors associated with insomnia among adolescents in northern Peru in a post-pandemic scenario.

Although data collection was conducted in 2022, the present study captures a critical post-pandemic transitional period rather than the acute phase of the COVID-19 crisis. Emerging evidence suggests that the psychosocial, behavioral, and mental health consequences of the pandemic—particularly among adolescents—may persist over time, even as infection rates decline. Sleep disturbances, changes in technology use, and exposure to pandemic-related stressors may continue to shape adolescent well-being beyond the immediate context of COVID-19.

Therefore, the objective of this study was to determine the prevalence and factors associated with insomnia in adolescents from five schools in northern Peru.

## 2. Materials and Methods

### 2.1. Study Design

A cross-sectional, secondary data analysis study was conducted on adolescents from five educational institutions in the Lambayeque region. The objective was to determine the prevalence and factors associated with insomnia. The primary study aimed to evaluate the association between acne and mental health outcomes.

### 2.2. Population and Sample

The target population comprised 1972 students enrolled in five secondary schools located in the Lambayeque region of Peru. The original data collection was carried out between 13 September and 15 December 2022.

In the primary study, a total of 1442 adolescents met the eligibility criteria and were included in the initial analytical sample. Eligible participants were students who attended school regularly and completed the main study questionnaires. Adolescents whose parents or legal guardians did not provide informed consent, as well as those who failed to provide informed assent or did not complete the questionnaires, were excluded. For the present secondary data analysis, an additional 129 records were excluded due to incomplete information on the insomnia-related variables. A non-probability sampling approach was used. The final sample for the secondary analysis consisted of 1313 adolescents, corresponding to a participation rate of 66.6%.

Statistical power was estimated at 97.9% to identify differences between a relative who died of COVID and insomnia. The proportion of insomnia in the group without a relative who died of COVID-19 (p1 = 0.334) and in the group with a relative who died of COVID-19 (p2 = 0.442) was used. Additionally, the following sample size was used: n1 = 734 for the group of adolescents without a relative who died of COVID-19 and n2 = 579 for the group of adolescents with a relative who died of COVID-19. Similarly, the same procedure was performed for the self-esteem variable, resulting in a statistical power of 100%. The proportion of insomnia in adolescents without low self-esteem (p1 = 0.311) and with low self-esteem (p2 = 0.482) was used, along with their respective sample sizes (n1 = 726, group without low self-esteem; and n2 = 587, group with low self-esteem).

### 2.3. Procedures

#### 2.3.1. Variables and Instruments

The primary outcome was insomnia, defined using the total score of the Insomnia Severity Index (ISI). Scores were obtained by summing all questionnaire items, with values > 7 indicating the presence of insomnia. Insomnia severity was classified as no clinical insomnia (0–7 points), subclinical insomnia (8–14 points), moderate clinical insomnia (15–21 points), and severe clinical insomnia (22–28 points). For analytical purposes, this variable was subsequently dichotomized into absence of insomnia (0–7 points) and presence of insomnia (>7 points). Secondary independent variables included family functioning, assessed using the Family APGAR and categorized as normal (17–20 points), mild dysfunction (13–16 points), moderate dysfunction (10–12 points), and severe dysfunction (0–9 points). Self-esteem was evaluated using the Rosenberg Self-Esteem Scale and classified as high (30–40 points), moderate (26–29 points), or low (0–25 points); this variable was further dichotomized into average–high versus low self-esteem. Risk of eating disorders was determined using the SCOFF questionnaire, with a score of ≥2 indicating a positive screening result. Facial acne severity was assessed according to a standardized acne severity scale, ranging from grade 1 to grade 4. Resilience was measured using the abbreviated version of the Connor–Davidson Resilience Scale (CD-RISC), with scores categorized as low (0–29 points) or high (≥30 points). Exposure to bullying was defined as a score of ≥2 points based on the sum of responses to the seven items of the European Bullying Intervention Project Questionnaire (EBIPQ). Finally, acne-related quality of life was assessed using the Dermatology Life Quality Index (DLQI) and categorized as no effect (0–1 points), small effect (2–5 points), moderate effect (6–10 points), large effect (11–20 points), or extremely large effect (21–30 points).

Additionally, a comprehensive set of socio-educational, academic, and psychosocial variables was collected. Age was recorded in years and categorized by adolescent developmental stage (early, middle, and late). Sex was assessed using a binary classification (male/female), as defined in the original questionnaire. Other variables included type of educational institution (public or private), area of residence (rural, urban, or peri-urban), household size (1–5, 6–10, or 11–15 members), religious affiliation (none, Catholic, or non-Catholic), and family history of mental disorders (yes/no). Nutritional status was determined using body mass index categories (underweight, normal weight, overweight, and obesity). Social and interpersonal characteristics were evaluated through self-reported closeness with friends and relatives (rare, frequent, or very frequent). Academic and relational factors included having failed at least one school subject (yes/no) and having a romantic partner (yes/no). Substance use behaviors were assessed through self-reported alcohol intake (never, monthly, 2–4 times per month, 2–3 times per week, or ≥4 times per week) and cigarette smoking (never, <10 cigarettes/day, 11–20 cigarettes/day, 21–30 cigarettes/day, or ≥31 cigarettes/day). Additional variables captured psychosocial and behavioral aspects, including seeking mental health support during the COVID-19 pandemic (yes/no), frequency of social media use (none, low, moderate, high, or extreme), daily internet use (1–5, 6–10, or 11–15 h/day), daily television viewing time (1–5, 6–10, or 11–15 h/day), and experiencing the death of a family member due to COVID-19 during the pandemic (yes/no).

#### 2.3.2. Insomnia (ISI)

An instrument that uses a Likert-type scale to assess the impact of insomnia. It consists of a total of 7 items with possible responses ranging from “none” (0 points) to “very severe” (4 points), with a higher score correlating with greater severity of insomnia. A score greater than 7 has been established as the cutoff point to identify a person with insomnia. The results are determined based on the score as absence of clinical insomnia (0–7 points), subclinical insomnia (8–14 points), moderate clinical insomnia (15–21 points), and severe clinical insomnia (22–28 points) [26]. It has adequate convergent validity and reliability indices greater than 0.80 [27]. This instrument has been used to evaluate Latino adolescents, especially in the Mexican and Salvadoran populations, finding Cronbach’s alphas that fluctuate between 0.82 and 0.86 [26,27]. It was previously used to evaluate this adolescent population during the COVID-19 pandemic [8,14].

#### 2.3.3. Family Dysfunction (Family APGAR)

An instrument that uses a Likert-type scale to assess family functioning. It consists of a total of 5 items with possible responses ranging from “never” (0 points) to “always” (4 points), where a lower score correlates with greater family dysfunction. A score of 16 or less has been established as the cutoff point to identify family dysfunction. The results are determined based on the score as normal (17–20 points), mild dysfunction (16–13 points), moderate dysfunction (12–10 points), and severe dysfunction (0–9 points) [28]. It has adequate internal consistency (α = 0.79) [29]. This instrument has been used to evaluate Latino adolescents, particularly in the Colombian population, finding Cronbach’s alphas between 0.89 and 0.91 [30]. It has also been used in adolescents in the context of the COVID-19 pandemic [31,32].

#### 2.3.4. Self-Esteem (Rosemberg)

An instrument that uses a Likert-type scale to assess personal self-esteem. It consists of a total of 10 items, 5 positively worded and 5 negatively worded, with possible responses ranging from “strongly disagree” (0 points) to “strongly agree” (4 points), with a lower score correlating with lower personal satisfaction. The results are determined based on the score as high self-esteem (30–40 points), average self-esteem (26–29 points), and low self-esteem (0–25 points). It has adequate internal consistency (alpha = 0.80) [33]. In addition, it has been useful in assessing self-esteem in Latino adolescents, especially in the Ecuadorian population, with Cronbach’s alphas between 0.71 and 0.86 [34]; and also during the pandemic caused by COVID-19 [35,36].

#### 2.3.5. Eating Disorder (SCOFF)

An instrument used to assess the risk of eating disorders. It consists of a total of 5 items with possible answers of “yes” or “no.” Two or more affirmative responses have been established as the cutoff point to identify a high probability of anorexia or bulimia nervosa [37]. It has adequate internal consistency (α = 0.52) [38]. This instrument was used to evaluate Latino adolescents, particularly Colombians, finding Cronbach’s alphas that fluctuate between 0.44 and 0.48 [39,40]. In addition, the instrument was also used during the waves of the COVID-19 pandemic [41,42].

#### 2.3.6. Facial Acne (Spanish Acne Severity Scale)

An instrument that uses a visual scale to determine acne severity. It consists of a series of images ranked by severity: on the face (4 photos), the chest (3 photos), and the back (3 photos). These are classified from grade 1 (least severe) to grade 4 (most severe) [43]. Lesions are counted and classified as non-inflammatory, superficial inflammatory, deep inflammatory and residual [44]. It has adequate internal consistency (α = 0.52) [45]. During the COVID-19 pandemic, this scale was also designed to assess adolescents in this context [45,46].

#### 2.3.7. Resilience (Abbreviated CD-RISC)

An instrument that uses a Likert-type scale to measure resilience. It consists of a total of 10 items with possible responses: “always” (4 points), “almost always” (3 points), “sometimes” (2 points), “rarely” (1 point), and “never” (0 points). A higher score correlates with greater resilience. The response time is also 10 to 15 min [47]. It has adequate validity and a high reliability index due to internal consistency (α = 0.85) [47]. This instrument has also been used in adolescents from Metropolitan Lima, resulting in Cronbach’s alpha coefficients of 0.83 [47]. Likewise, the instrument has been used in adolescents and university students in the context of the COVID-19 pandemic [48,49].

#### 2.3.8. Bullying (European Bullying Intervention Project Questionnaire)

We used an instrument that uses a Likert-type scale to measure the frequency of face-to-face bullying. It consists of a total of 14 items with possible responses: “never” (0 points), “once or twice” (1 point), “once or twice a month” (2 points), “about once a week” (3 points), and “more than once a week” (4 points). The first 7 items are about victimization and the last 7 are about aggression [50]. The results are determined according to the score as victim role (2 or more points in victimization items and 1 or less in aggression items), aggressor (2 or more points in aggression items and 1 or less in victimization items), and aggressor-victimized (2 or more points in at least the aggression and victimization items) [51]. It has adequate validity and reliability indexes of 0.72 [50]. It has been used to evaluate Latino adolescents, especially in the Peruvian population, finding Cronbach’s alphas of 0.77 [52]. It has also been used on adolescents during the COVID-19 pandemic [53,54].

#### 2.3.9. Quality of Life (DLQI Instrument)

We used an instrument that uses a Likert-type scale to assess quality of life in dermatology. It consists of a total of 10 items with possible responses: “not at all/not relevant” (0 points), “a little” (1 point), “a lot” (2 points), and “very much” (3 points), where a higher score correlates with a lower quality of life. The results are determined based on the score as: does not affect the patient’s life at all (0 to 1 point); small effect on the patient’s life (2 to 5 points); moderate effect on the patient’s life (6 to 10 points); large effect on the patient’s life (11 to 20 points); extremely large effect on the patient’s life (21 to 30 points) [55]. It has adequate validity and has been applied to Latin American adolescents, finding, in the Mexican population, Cronbach’s alphas of 0.85 [56]. In the context of COVID-19, this assessment has also been used in adolescents [45,57].

Internal consistency in the present sample was assessed using Cronbach’s alpha coefficients. All instruments showed good to excellent reliability, including the Insomnia Severity Index (α = 0.83), Rosenberg Self-Esteem Scale (α = 0.86), Family APGAR (α = 0.93), Connor–Davidson Resilience Scale (α = 0.94), Dermatology Life Quality Index (α = 0.94), European Bullying Intervention Project Questionnaire (α = 0.88), and the Spanish acne severity scale (α = 0.78). The SCOFF questionnaire showed lower internal consistency (α ≈ 0.6), consistent with its use as a brief screening instrument in adolescent populations; therefore, it was retained due to its validated application in large epidemiological studies [58].

### 2.4. Statistical Analysis Plan

All statistical analyses were conducted using Stata version 17.0 (StataCorp, College Station, TX, USA).

Categorical variables were summarized using absolute and relative frequencies, while continuous variables (e.g., age) were described using appropriate measures of central tendency and variability, selected after assessing data distribution normality.

Associations between insomnia and explanatory variables were initially explored through bivariate analyses. The chi-square test was applied when assumptions regarding expected cell counts were met. To estimate factors independently associated with insomnia, both unadjusted and adjusted regression analyses were performed. Generalized linear models with a Poisson distribution, log link function, and robust variance estimators were used, accounting for clustering at the school level. Results were expressed as prevalence ratios (PRs) with corresponding 95% confidence intervals (95% CIs). Variables achieving statistical significance in the unadjusted analyses (*p* < 0.05) were included in the multivariable model, and potential multicollinearity among predictors was evaluated in the final adjusted model.

Covariate selection for the multivariable models was guided by an epidemiological framework informed by prior literature on adolescent insomnia and mental health. Variables with established theoretical relevance were retained in the adjusted models regardless of their statistical significance in bivariate analyses, while bivariate results were used as an exploratory step to describe crude associations rather than as the sole criterion for model inclusion.

Analyses were conducted using a complete-case approach for each model, whereby participants with missing data in the variables included in a given analysis were excluded from that specific model. Within the analytical sample, the proportion of missing data across psychosocial variables was less than 10%, and no data imputation procedures were applied.

### 2.5. Ethical Aspects

The study protocol received approval from the Ethics Committee of Universidad San Martín de Porres, Lima, Peru (Approval No. 348-CIEI-FMH-USMP). All research procedures were conducted in accordance with internationally recognized ethical guidelines, including the principles outlined in the Declaration of Helsinki. To protect participant privacy, data were collected using anonymized questionnaires. In addition, written informed consent was obtained from parents or legal guardians, and written assent was secured from all participating adolescents prior to data collection.

## 3. Results

### 3.1. Socio-Educational Characteristics of Adolescents

Of 1313 adolescents, 54.3% were male, and the mean age was 14.6 years (age range: 11–19 years). 12.9% were overweight. 20.9% reported seeking mental health help during the COVID-19 pandemic, 32.7% reported using social media extensively during the pandemic, while 22.8% reported using the internet between 6 and 10 h/day. 50.5% reported having had a family member hospitalized with COVID-19, and 44.1% reported that a family member had died due to the pandemic. The majority reported low self-esteem (44.7%), severe family dysfunction (29.8%), and low resilience (82.8%). 39.2% presented an eating disorder. 12.5% reported consuming alcohol monthly, while 3.2% reported smoking fewer than 10 cigarettes/day. The rest of the socio-educational-psychosocial variables are shown in Table 1.

### 3.2. Insomnia in Adolescents

**Figure 1:**
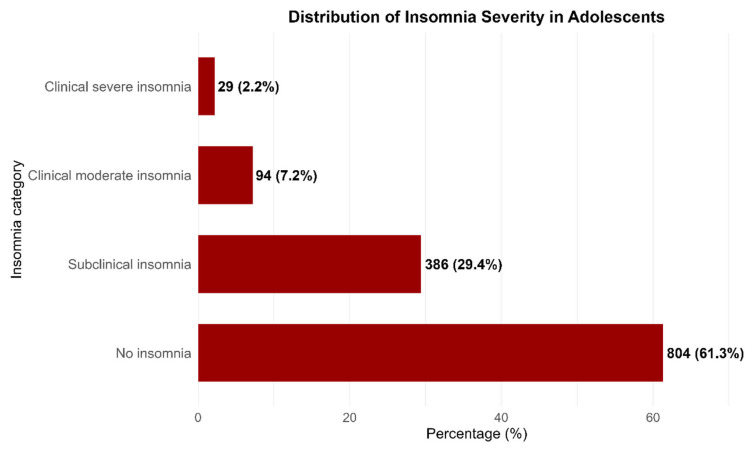
Distribution of Insomnia Severity in Adolescents.

The prevalence of insomnia in adolescents was 38.9% (95% CI: 36.12–41.46). Subclinical insomnia was present in 29.4%, moderate insomnia in 7.2%, and severe insomnia in 2.2%. Table 1 and Figure 1.

**Figure 2:**
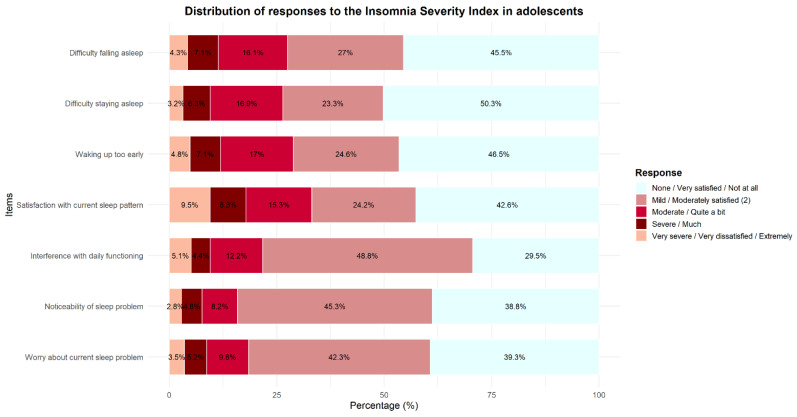
Distribution of responses to the Insomnia Severity Index in Adolescents.

Regarding insomnia symptoms, 16.1% of adolescents reported a moderate difficulty falling asleep, 16.8% reported a moderate difficulty maintaining sleep, and 17.0% reported moderately early morning awakenings. In addition, 9.5% reported being very dissatisfied with their current sleep pattern, 12.2% indicated that insomnia symptoms significantly interfered with daily functioning, and 9.7% reported being quite concerned about their current insomnia symptoms (Figure 2).

### 3.3. Factors Associated with Insomnia, in Bivariate Analysis

Students with a family history of mental health problems had a higher prevalence of insomnia (54.2% vs. 36.1%, *p* < 0.001). Students who had failed a course had a higher prevalence of insomnia (44.1% vs. 34.3%, *p* < 0.001). Students who consumed alcohol monthly or less had a higher prevalence of insomnia compared to those who had never consumed alcohol (49.4% vs. 35.9%, *p* = 0.001). Students who had sought mental health help at some point during the pandemic had a higher prevalence of insomnia (54.2% vs. 34.7%, *p* < 0.001). Students whose family members had died from COVID-19 had a higher prevalence of insomnia (44.2% vs. 34.5%, *p* < 0.001). Those with eating disorder symptoms had a higher prevalence of insomnia (57.7% vs. 27.1%, *p* < 0.001). Those with low self-esteem had a higher prevalence of insomnia compared to those with medium-high self-esteem (48.2% vs. 31.1%; *p* < 0.001). Additionally, a significant association was observed between the presence of insomnia and sex (*p* < 0.001), frequency of social media use (*p* < 0.001), amount of time spent on the internet (*p* < 0.001), and the impact of COVID-19 on life (*p* < 0.001). Table 2.

### 3.4. Factors Associated with Insomnia, in Simple and Multiple Regression Analysis

**Figure 3:**
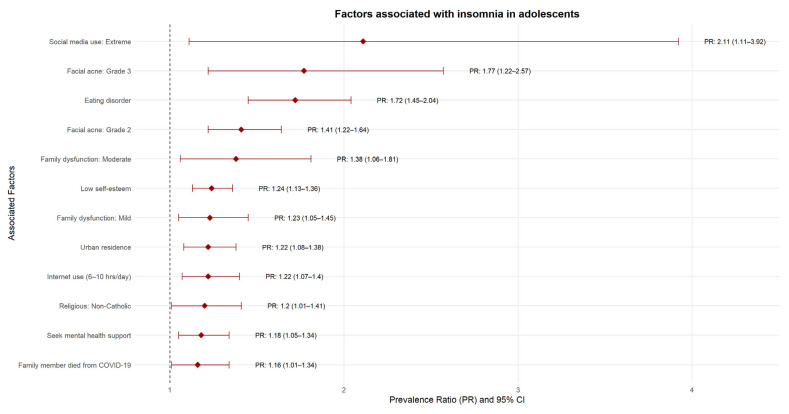
Factors associated with insomnia in Adolescents.

In multiple regression analysis, we found that residing in urban areas was associated with a 22% increase in the prevalence of insomnia compared to rural areas (OR: 1.22; 95% CI: 1.08–1.38). Belonging to a non-Catholic religion was associated with a 20% increase in the prevalence of insomnia (OR: 1.20; 95% CI: 1.01–1.41). Students who sought mental health help had an 18% higher prevalence of insomnia (OR: 1.18; 95% CI: 1.05–1.34). Excessive social media use increased the prevalence of insomnia by 111% (OR: 2.11; 95% CI: 1.11–3.92). Adolescents who reported having a family member die from COVID-19 during the pandemic had a 16% increased prevalence of insomnia (OR: 1.16; 95% CI: 1.01–1.34). Internet use between 6 and 10 h per day increased the prevalence of insomnia by 22% (OR: 1.22; 95% CI: 1.07–1.40). Mild and moderate family dysfunction increased the prevalence of insomnia by 23% (OR: 1.23; 95% CI: 1.05–1.45) and 38% (OR: 1.38; 95% CI: 1.06–1.81), respectively. Adolescents with low self-esteem had a 24% higher prevalence of insomnia (OR: 1.24; 95% CI: 1.13–1.36), while having an eating disorder increased this prevalence by 72% (OR: 1.72; 95% CI: 1.45–2.04). Adolescents with grade 2 and 3 acne on the face had a 41% (OR: 1.41; 95% CI: 1.22–1.64) and 77% (OR: 1.77; 95% CI: 1.22–2.57) higher prevalence of insomnia, respectively. Figure 3 and Table 3.

## 4. Discussion

### 4.1. Prevalence of Insomnia

In this study, just over one-third of adolescent schoolchildren (38.8%) experienced insomnia, with 7.2% and 2.2% presenting moderate and severe insomnia, respectively. This prevalence is comparable to findings reported in adolescents from other countries, including Canada (38.8%) [8], Sweden (48%) [59], and Peru (49%) [60], although higher and lower estimates have also been described in Colombia (76%) [11] and Peru (13%) [16], reflecting substantial heterogeneity across contexts. The relatively high prevalence observed may be understood within a broader explanatory framework involving increased psychosocial stress, anxiety, and disruptions to daily routines during and after the COVID-19 pandemic. Pandemic-related changes—such as school closures, distance learning, and irregular schedules—have been shown to alter sleep–wake patterns and contribute to sleep dysregulation among adolescents [61,62,63,64]. These findings suggest that adolescent insomnia should be viewed as a multifactorial phenomenon influenced by contextual stressors and behavioral changes, rather than as an isolated sleep problem.

### 4.2. Factors Associated with Insomnia

Adolescents living in urban areas were associated with a higher prevalence of insomnia. This is similar to what Amaral et al. (2013) reported, who, in their research on adolescents in Portugal, found that those living in urban areas were more likely to experience insomnia compared to those living in rural areas (OR: 1.35; 95% CI: 1.08–1.68) [5]. Zheng et al. (2018), who in their research on adults in China found that there was an association between the area of residence and the prevalence of insomnia, 22.9% of urban residents had insomnia, while 13.4% of rural residents had insomnia (X2 = 14.9, *p* < 0.001) [65]. It also correlates with the study by Guerrero-Zúñiga et al. (2018) conducted on young adults in Mexico, who found a higher probability of insomnia in urban residents (OR = 1.37) [66]. Herrera-Escobar et al. (2023), on the other hand, in their research on medical students at a university in northern Peru, found that there is no association between the type of residence, whether urban or rural, with the prevalence of insomnia (*p* < 0.129) [67]. The association found could be explained by a higher population density in urban areas, which is directly related to a higher proportion of COVID-19 cases and deaths in cities [68]. This could have caused greater stress and anxiety in adolescents, generating an increase in the prevalence of insomnia [69]. Another explanation could be due to the greater exposure to artificial light that occurs in urban areas, especially the light emitted by electronic devices, which interferes with the circadian rhythm and causes difficulty in falling asleep [70,71]. Finally, the changes in daily routine that adolescents experienced in urban areas were more drastic in the lifestyle they led [72,73], due to social restrictions imposed, for example, the closure of shopping centers and leisure clubs, which caused an increase in adolescent stress and contributed to the increase in insomnia [74,75].

Although sex was associated with insomnia in bivariate analyses, this association was attenuated after multivariable adjustment, suggesting that gender differences in insomnia may be partially mediated by psychosocial and behavioral factors such as technology use, emotional regulation, and social context [76]. Previous studies have reported important gender-specific patterns in both insomnia and problematic technology use during adolescence, often reflecting differences in emotional vulnerability, coping strategies, and online behaviors [77,78]. Future research using stratified analyses or interaction models is warranted to better elucidate gender-specific pathways underlying adolescent insomnia [76].

Professing a non-Catholic religion was associated with a higher prevalence of insomnia among adolescents in this study. Previous evidence on this association is mixed. While Fergason et al. (2020) reported differences in sleep quality and duration across religious affiliations in U.S. adolescents, with atheists/agnostics and Baptists showing better sleep outcomes than Catholics [79], Lopes-Rocha et al. (2002) found no significant association between religion and insomnia in Brazilian adults [80]. These findings suggest that religious affiliation per se may not directly determine sleep quality, but rather that its relationship with insomnia is context-dependent and shaped by broader social and cultural factors. During the COVID-19 pandemic, religious beliefs and practices were shown to provide emotional comfort, social support, and coping resources that may have mitigated stress, anxiety, and grief [81,82,83].

In adolescents, religion can offer values, social support, and a sense of belonging—particularly through participation in youth groups—which may promote emotional regulation and reduce anxiety [84,85]. However, in the Peruvian sociocultural context, where Catholicism predominates, adolescents from minority religious groups may face reduced social integration or fewer community-based support networks. These contextual stressors, rather than religiosity itself, may help explain the observed association with insomnia. Overall, this finding likely reflects broader social mechanisms related to religious minority status, underscoring the need for future research on the interplay between religious affiliation, perceived belonging, and adolescent sleep health.

Adolescents who reported seeking mental health support during the COVID-19 pandemic were associated with a higher prevalence of insomnia. This is consistent with a study of adolescents in Taiwan, which found that depression (β = 0.29) and anxiety (β = 0.14) were positively associated with insomnia [86]. It is also associated with the study conducted on medical students in Peru, where the highest prevalence of insomnia was found in those with depressive symptoms during the COVID-19 pandemic (PR = 2.49) [87]. Additionally, it is associated with what was reported in Peru, where medical university students presented a higher prevalence of insomnia in those who had mental health symptoms during the COVID-19 pandemic (RPa = 10.48) [88]. This correlates with the study by Zachrisson et al. (2006), who, in their research conducted on adolescents in Norway, found that 34% of students who had mental health problems sought professional help [89]. It also aligns with what was reported by Essau (2005), who conducted a study on secondary school students in Germany and found that 18.2% of students who had mental health problems sought help [90]. This association could be explained by the considerable impact that the pandemic has had on the mental health of adolescents due to factors such as stress, anxiety, uncertainty, social isolation and changes in daily routine. These factors contribute to the development of mental health problems, which, in turn, may be related to insomnia [91,92]. Furthermore, insomnia and mental health problems can feed off each other in a negative cycle [93]. Lack of adequate sleep can increase emotional vulnerability and the risk of mental health problems, and in turn, mental health problems can contribute to insomnia by generating negative thoughts, worries or difficulties relaxing and falling asleep [93,94].

Reporting extreme social media use was associated with a higher prevalence of insomnia among adolescents. This is similar to what Woods et al. (2016) reported, who in their study of Scottish adolescents found a positive association between insomnia and social media use (r = 0.24, *p* < 0.001) [95]. Ozgun et al. (2016) found a positive association between these two variables in adolescents from Türkiye (OR: 1.81; 95% CI: 1.36–2.39) [96]. It also correlates with the findings of Cornejo-Canaza et al. (2022), who, in their research on adolescent schoolchildren from a school in southern Peru, found a significant association between social media addiction and sleep quality (X2 = 16.982, *p* = 0.009), concluding that these adolescents may have insomnia [97]. However, this contrasts with the study by Nursalam et al. (2019), who found no association between insomnia and social media use in Indonesian adolescents (*p* = 0.965) [98]. This association could be explained because the use of social networks involves interacting with visual, auditory and textual content that generates intense mental stimulation [99]. The use of electronic devices and exposure to bright light from screens can interfere with the production of melatonin, a hormone that regulates the sleep–wake cycle, making it difficult to fall asleep [100]. Additionally, on social media, teenagers constantly compare themselves with others, which can generate anxiety and stress. Added to this is the social pressure to be constantly connected, have a perfect image or receive online recognition, which can increase anxiety levels and make it difficult to relax, which is necessary to fall asleep, generating insomnia [95].

Having a family member who died from COVID-19 was associated with a higher prevalence of insomnia among adolescents in this study. Evidence on this association has been inconsistent. While Brown et al. (2023) reported a positive relationship between family exposure to COVID-19 and insomnia [101], other studies found no significant associations in different populations, including adolescents and medical students [87,102]. These discrepancies suggest that the impact of family loss due to COVID-19 on sleep may depend on contextual and individual factors. From a psychosocial perspective, bereavement represents a complex stressor during adolescence, a developmental stage characterized by heightened emotional vulnerability. Grief reactions—such as emotional distress, intrusive thoughts, fear of further losses, and feelings of guilt—may interfere with sleep initiation and maintenance, thereby increasing the risk of insomnia [103,104,105]. In this context, the observed association likely reflects the role of cumulative emotional stress and unresolved grief rather than exposure to COVID-19 infection itself. This interpretation supports the need for psychosocial support strategies that address grief and emotional regulation as part of sleep health promotion in adolescents.

Adolescents who reported 6 to 10 h of daily internet use were associated with a higher prevalence of insomnia. This finding is consistent with previous studies conducted in different settings, which have shown that excessive internet use—particularly during evening or nighttime hours—is strongly associated with sleep disturbances among adolescents [10,106,107]. From an integrative perspective, prolonged internet use may disrupt sleep through multiple mechanisms, including delayed bedtimes, interference with regular sleep schedules, and circadian rhythm dysregulation. In addition, heightened cognitive and emotional arousal related to online activities—such as social media engagement, gaming, or exposure to emotionally charged content—may further impair sleep initiation and maintenance [108,109]. These mechanisms may have been exacerbated during the COVID-19 pandemic, when increased screen time and exposure to stressful or negative information intensified emotional distress and sleep difficulties among adolescents [110]. However, the persistence of high internet use in post-pandemic contexts suggests that this association reflects broader behavioral patterns that continue to influence adolescent sleep health beyond the acute pandemic period. Beyond individual associations, the findings can be interpreted within an integrative framework linking problematic technology use, emotional regulation [111], prosocial functioning [112], and sleep health in adolescents [113]. Excessive use of the internet and social media may contribute to emotional dysregulation through heightened cognitive and emotional arousal, exposure to social comparison, and disrupted circadian rhythms. In turn, emotional dysregulation may reduce adolescents’ capacity for adaptive coping and prosocial engagement, potentially weakening protective social bonds and increasing vulnerability to sleep disturbances. Conversely, impaired sleep may further exacerbate emotional dysregulation and maladaptive technology use, creating a bidirectional and self-reinforcing cycle. This integrative perspective highlights the complex interplay between behavioral, emotional, and social factors underlying adolescent insomnia and underscores the need for multifaceted prevention and intervention strategies [114,115].

Recent literature provides a broader conceptual framework to interpret the strong associations observed between technology use and insomnia in adolescents [116]. Studies on problematic internet and smartphone use have consistently shown gender-specific patterns, with differences in usage motives, emotional vulnerability, and associated psychological outcomes [117]. Moreover, mediation models suggest that emotional dysregulation may partially explain the relationship between excessive social media or internet use and adverse mental health outcomes, including sleep disturbances [118].

In addition, research on bedtime procrastination highlights how problematic smartphone use can delay sleep onset through heightened cognitive arousal and impaired self-regulation, further compromising sleep quality [119]. Importantly, resilience and prosocial functioning have been identified as protective factors that may buffer the negative impact of excessive technology use by enhancing adaptive coping, emotional regulation, and social connectedness [120]. Integrating these perspectives supports a multidimensional interpretation of adolescent insomnia, emphasizing the interplay between behavioral patterns, emotional processes, and psychosocial resources without detracting from the main focus of the present study [116].

Adolescents with mild and moderate family dysfunction were associated with a 23% and 38% higher prevalence of insomnia, respectively. This result is similar to that reported by Chang et al. (2019), who in their research on adolescent schoolchildren in Taiwan found that family dysfunction was negatively associated with adolescent sleep quality (r = −0.08; 95% CI −0.03, −0.13; *p* < 0.001) [121]. It also correlates with what was reported by Brodar et al. (2020), who studied adolescents starting high school in the United States and found that family functionality was negatively related to insomnia symptoms (r = −0.14; 95% CI: −0.16, −0.32; *p* < 0.001) [122]. However, this is contrary to what was described by Wang et al. (2020), who, in their study in five secondary schools in the United States, found that there is no association between insomnia and family functionality (r = 0.01; 95% CI: −0.11 to 0.13; *p* = 0.208) [123]. This association could be explained by the frequent conflicts that family dysfunction implies due to the presence of problems and tensions among family members, including domestic violence and emotional abuse [124]. It could also be due to the perception of insecurity and lack of emotional stability due to communication problems and conflicts that occur within dysfunctional families [124]. Lack of a safe and loving family environment can lead to anxiety, worry and fear [125]. These factors create a stressful environment for the teenager, which can make it difficult to fall asleep [121,126] and maintain restful sleep [127,128].

Adolescents with low self-esteem were associated with a higher prevalence of insomnia. This is similar to what Woods et al. (2016) reported, who in their study of Scottish adolescents found that insomnia was associated with lower self-esteem (r = −0.41, *p* < 0.001) [95]. Likewise, it correlates with what was found by Heitun et al. (2020) who in their study on Norwegian high school students reported an association between the seven dimensions of self-esteem (a) scholastic competence (*p* = 0.013); (b) social acceptance (*p* < 0.001); (c) athletic competence (*p* = 0.001); (d) physical appearance (*p* = 0.001); (e) romantic attraction (*p* = 0.038); (f) close friendship (*p* = 0.026); perceived self-esteem (*p* < 0.001); when compared with insomnia [129]. This association could be explained by the excessive worry and anxiety that adolescents with low self-esteem may experience because they often have a negative view of themselves and may experience constant worries about their appearance, skills, abilities, or personal worth [130]. This excessive worry and associated anxiety can make it difficult to relax, which is necessary for sleep [95]. Low self-esteem can be both a cause and a consequence of insomnia, and a lack of restful sleep can further affect a teenager’s self-esteem and emotional well-being, creating a negative cycle [95].

Having an eating disorder (ED) was associated with a higher prevalence of insomnia. This is similar to what Nagata et al. (2021) reported, who, in their seven-year longitudinal study of young adults in the United States, found a higher incidence of sleep disturbances, including insomnia, in people with eating disorders (IRR: 1.24; 95% CI: 1.05–1.46; *p* < 0.001) [131]. It also agrees with what was found by Reyna-Culquitante (2020), who, in his study of Peruvian university students, found a prevalence of 64% of students with insomnia and eating disorders; likewise, he found a higher probability of insomnia in students with eating disorders (OR = 4.81; 95% CI: 2.10–7.90; *p* < 0.005) [132]. However, this is contrary to what was reported by Beigrezaei et al. (2022) who studied adolescents from Iran and found no significant association between insomnia and eating disorders (*p* = 0.233) [133]. The association could be explained due to the relationship that eating disorders have with other mental health problems, including depression, anxiety or post-traumatic stress disorder, in addition to generating obsessive concerns about food, weight and body image, which leads to intrusive and persistent thoughts [132,134]. These disorders can cause symptoms of insomnia, such as difficulty falling asleep, frequent awakenings during the night, or fragmented sleep [135].

Presenting grade 2 and grade 3 facial acne severity was associated with a 41% and 77% higher prevalence of insomnia, respectively. This is similar to what Schrom et al. (2019) reported, who studied young adults in the United States and found a positive relationship between acne severity and insomnia (r = 0.483, *p* = 0.001) [136]. Likewise, it is similar to that reported by Harlim et al. (2020) who in their study in adolescents and young adults in Indonesia found a positive association between insomnia and the severity of acne vulgaris (*p* < 0.05) [137]. However, this contrasts with the study by Lim et al. (2022) who, in their research on high school and university students in Malaysia, found no association between insomnia and acne severity (*p* = 0.884) [138]. This association could be explained by the significant emotional impact that severe acne can have on the adolescent’s self-esteem and confidence, which causes social anxiety [139]. People with severe acne may feel self-conscious or embarrassed about their appearance, causing constant concern about how they are perceived by others [140]. These worries, feelings, and emotional stress can interfere with sleep, making it difficult to relax, which leads to insomnia [136].

Recent studies published after 2023 continue to report high prevalence of sleep disturbances among adolescents and emphasize the role of problematic technology use, emotional dysregulation, and persistent psychosocial stressors in post-pandemic contexts [141,142,143].

Although the COVID-19 pandemic no longer represents an acute public health emergency, many of the behavioral and psychosocial patterns identified in this study remain highly relevant in current post-pandemic contexts. Changes in technology use, sleep routines, emotional regulation, and social interactions that intensified during the pandemic may have become entrenched habits among adolescents. Therefore, the present findings contribute to understanding ongoing vulnerabilities in adolescent sleep health and can inform contemporary prevention and mental health promotion strategies beyond the immediate COVID-19 period.

Given the cross-sectional design of this study, the associations identified should not be interpreted as evidence of causal relationships. Rather, the findings reflect correlational patterns between insomnia and the examined sociodemographic, psychosocial, behavioral, and health-related factors.

### 4.3. Relevance of Findings in Mental Health

This study is relevant because it examines the prevalence of insomnia and its associated factors in adolescents, a population undergoing critical developmental transitions during which behavioral and lifestyle patterns are established and may persist into adulthood [144,145]. The findings highlight how disruptions in daily routines, social environments, and psychosocial stressors—intensified during the COVID-19 pandemic—have affected adolescent sleep health. By identifying factors associated with insomnia, this study provides evidence to raise awareness about the importance of healthy sleep and its implications for physical, emotional, and academic well-being [94,146,147]. Moreover, these findings support the development of targeted, evidence-based strategies to promote healthy sleep and mental well-being among adolescents in both clinical and school-based settings [148,149].

The findings of this study have several practical implications for adolescent mental health promotion and insomnia prevention. First, school-based programs should incorporate sleep health education as part of comprehensive mental health curricula, emphasizing regular sleep schedules, sleep hygiene, and the recognition of insomnia symptoms [150]. Second, psychoeducational interventions targeting the responsible use of digital technologies are warranted, given the strong associations observed between internet and social media use and insomnia. Such interventions should address emotional regulation, bedtime routines, and strategies to reduce excessive screen exposure, particularly before sleep [151,152]. Finally, preventive strategies aimed at strengthening self-esteem, resilience, and prosocial behaviors may play a protective role against insomnia. Interventions that enhance adaptive coping skills, emotional regulation, and supportive peer and family relationships could mitigate vulnerability to sleep disturbances and promote overall adolescent well-being [153].

### 4.4. Limitations and Strengths

Several limitations should be considered when interpreting the findings of this study. First, the cross-sectional design precludes the establishment of causal relationships between insomnia and the associated sociodemographic, psychosocial, behavioral, and health-related factors. Additionally, the prevalence and correlations of insomnia may vary across different stages of the COVID-19 pandemic, depending on fluctuations in infection rates, mortality, and related social restrictions. Second, the use of a non-probability sampling strategy limits the generalizability of the findings beyond participating in educational institutions. Third, as this study represents a secondary analysis of previously collected data, the authors had limited control over the measurement and quality assurance of certain variables. Consequently, some potentially relevant confounders—such as loneliness [8], family income [8], prior medical conditions [8], and coffee consumption [149]—were not available for inclusion, which may have resulted in residual confounding. Fourth, all variables were assessed through adolescent self-reports, which may be subject to information bias, including recall and social desirability bias.

This study represents a secondary analysis of data originally collected for an acne-focused investigation. As such, the study design and variable selection were not specifically tailored to insomnia research, which may have introduced selection bias and limited the availability of certain sleep-related confounders. Variables such as detailed sleep hygiene practices, caffeine consumption, loneliness, or preexisting sleep disorders were not assessed and may have influenced the observed associations. Consequently, residual confounding cannot be ruled out, and the findings should be interpreted with caution.

Despite these limitations, this study has notable strengths. It includes a large sample of adolescents from multiple educational institutions, enhancing internal validity. Moreover, the study integrates a wide range of psychosocial, behavioral, and mental health variables—rarely examined simultaneously in adolescent sleep research—providing a comprehensive epidemiological perspective on insomnia in a post-pandemic Latin American context.

Additionally, some screening instruments used in this study, particularly the SCOFF questionnaire, showed lower internal consistency compared with other measures. Although these instruments have been widely validated and used in adolescent populations, their modest reliability may have introduced non-differential measurement error, potentially attenuating some of the observed associations. Furthermore, the dichotomization of originally continuous variables, including insomnia severity and self-esteem, may have led to a loss of information and reduced analytical sensitivity. However, this approach was adopted to enhance clinical interpretability and comparability with previous epidemiological studies conducted in similar populations.

The temporal context of the study should also be considered. Although the data were collected in 2022, the findings remain relevant as they reflect the enduring impact of pandemic-related stressors during a post-pandemic period. Experiences such as the loss of a family member due to COVID-19 may function as markers of cumulative stress exposure rather than indicators of ongoing viral transmission. Therefore, the associations observed likely reflect longer-term vulnerabilities in adolescent sleep health rather than transient effects limited to the peak of the pandemic.

Finally, potential interaction effects—such as those by gender or stage of adolescence—were not explored in the present analyses. Although such effect modification may be relevant in the context of adolescent sleep and mental health, the primary aim of this study was to assess overall associations at the population level. Future studies with a priori hypotheses and sufficient statistical power should examine potential interactions to better understand subgroup-specific patterns of insomnia.

However, this research stands out as a pioneer in exploring the factors associated with insomnia in Peruvian adolescents during the pandemic, providing a valuable snapshot of this population’s mental health. The strength of using instruments with strong psychometric properties to ensure the validity and reliability of the measurements is highlighted. The large and diverse sample of adolescents met in person at their schools strengthens representativeness and lays a solid foundation for future research that thoroughly explores the explanatory factors of insomnia in adolescents, especially in the context of health emergencies such as epidemics or pandemics.

## 5. Conclusions

It was found that nearly 4 out of 10 adolescent participants during the COVID-19 pandemic presented with insomnia problems, associated with various socio-educational and psychosocial factors. Family dysfunction, low self-esteem, eating disorders, having a relative die from COVID-19, frequent internet and social media use, seeking mental health help, and the presence of acne were identified as factors linked to a higher prevalence of insomnia. These findings underscore the need for comprehensive interventions that address adolescent mental health in the context of the pandemic.

It is crucial to conduct educational campaigns targeting students, parents, and educators, promoting healthy sleep habits. Interdisciplinary collaboration between health professionals, educators, and communities is essential to develop effective approaches. Furthermore, the need for continued research is emphasized to better understand the effects of crises such as the pandemic on adolescent mental health and develop targeted intervention strategies.

## Figures and Tables

**Table 1:** Characteristics of schoolchildren from five schools in northern Peru.

Characteristics	n (%)
Age (years) *	14.6 ± 1.40
**Adolescent, according to stage**	
Early	299 (22.8)
Average	908 (69.2)
Late	106 (8.1)
**Sex**	
Female	600 (45.7)
Male	713 (54.3)
**Type of institution**	
National	857 (65.3)
Particular	456 (34.7)
**Place of residence**	
Rural	186 (14.2)
Urban	1092 (83.2)
Marginal urban	35 (2.7)
**Number of members in your family (categorized)**	
1 to 5	788 (60.0)
6 to 10	476 (36.3)
11 to 15	49 (3.7)
**Religion**	
Any	308 (23.5)
Catholic	741 (56.4)
Not Catholic	264 (20.1)
**Family mental history**	
No	1121 (85.4)
Yes	192 (14.6)
**Categorized BMI**	
Underweight	277 (21.1)
Normal	826 (62.9)
Overweight	169 (12.9)
Obesity	41 (3.1)
**Getting closer to friends**	
Infrequent	315 (24.0)
Frequent	616 (46.9)
Very common	382 (29.1)
**Approach with relatives**	
Infrequent	408 (31.1)
Frequent	593 (45.2)
Very common	312 (23.8)
**Failed course during school stage**	
No	714 (54.4)
Yes	599 (45.6)
**In love**	
No	492 (37.5)
Yes	821 (62.5)
**Alcohol consumption**	
Never	1027 (78.2)
Monthly or less	164 (12.5)
2 to 4 times a month	83 (6.3)
2 to 3 times a week	25 (1.9)
4 or more times a week	14 (1.1)
**Cigarette smoking**	
Never	1233 (93.9)
<10 cigarettes/day	42 (3.2)
11 to 20 cigarettes/day	23 (1.8)
21 to 30 cigarettes/day	6 (0.5)
31 or more cigarettes/day	9 (0.7)
**Seek mental health support**	
No	1038 (79.1)
Yes	275 (20.9)
**Frequency of social media use during the COVID-19 pandemic**	
Never	115 (8.8)
A bit	288 (21.9)
Moderate	363 (27.7)
A lot	429 (32.7)
Extreme	118 (9.0)
**Frequency of daily internet use**	
1 to 5	810 (61.7)
6 to 10	299 (22.8)
11 to 15	204 (15.5)
**Frequency of daily television use**	
1 to 5	1213 (92.4)
6 to 10	71 (5.4)
11 to 15	29 (2.2)
**Family member died from COVID-19**	
No	734 (55.9)
Yes	579 (44.1)
**Acne on face**	
No	669 (51.0)
Grade 1	559 (42.6)
Grade 2	60 (4.6)
Grade 3	7 (0.5)
Grade 4	18 (1.4)
Family dysfunction **	
No	493 (40.4)
Mild	241 (19.8)
Moderate	122 (10.0)
Severe	363 (29.8)
Resilience **	
Low	1080 (82.8)
High	225 (17.2)
**Victim of Bullying**	
No	851 (64.8)
Yes	462 (35.2)
Quality of life due to acne **	
It doesn’t affect anything-small	1053 (84.2)
Moderate-extreme effect	198 (15.8)
Eating disorder **	
No	752 (60.8)
Yes	485 (39.2)
**Self-esteem**	
High/Medium	726 (55.3)
Low	587 (44.7)
**Insomnia**	
No	804 (61.3)
Subclinical	386 (29.4)
Moderate clinical	94 (7.2)
Clinically severe	29 (2.2)

* Mean and standard deviation, ** Does not add up to 1313 due to missing data.

**Table 2:** Factors associated with insomnia in schoolchildren from five schools in northern Peru, bivariate analysis.

Variables	Insomnia		*p* *
	No (n = 804)	Yes (n = 509)	
	n (%)	n (%)	
**Adolescent, according to stage**			0.603
Early	186 (62.2)	113 (37.8)	
Average	549 (60.5)	359 (39.5)	
Late	69 (65.1)	37 (34.9)	
**Sex**			**<0.001**
Female	327 (54.5)	273 (45.5)	
Male	477 (66.9)	236 (33.1)	
**Type of institution**			0.833
National	523 (61.0)	334 (39.0)	
Particular	281 (61.6)	175 (38.4)	
**Place of residence**			0.317
Rural	121 (65.1)	65 (35.0)	
Urban	659 (60.4)	433 (39.7)	
Marginal urban	24 (68.6)	11 (31.4)	
**Number of members in your family (categorized)**			0.635
1 to 5	490 (62.2)	298 (37.9)	
6 to 10	286 (60.1)	190 (39.9)	
11 to 15	28 (57.1)	21 (42.9)	
**Religion**			0.118
Any	204 (66.2)	104 (33.4)	
Catholic	441 (59.5)	300 (40.5)	
Not Catholic	159 (60.2)	105 (39.8)	
**Family mental history**			**<0.001**
No	716 (63.9)	405 (36.1)	
Yes	88 (45.8)	104 (54.2)	
**Categorized BMI**			0.895
Underweight	167 (60.3)	110 (39.7)	
Normal	512 (62.0)	314 (38.1)	
Overweight	100 (59.2)	69 (40.8)	
Obesity	25 (61.0)	16 (39.0)	
**Getting closer to friends**			0.293
Infrequent	187 (59.4)	126 (40.6)	
Frequent	391 (63.5)	225 (36.5)	
Very common	226 (59.2)	156 (40.8)	
**Approach with relatives**			**<0.001**
Infrequent	210 (51.5)	198 (48.5)	
Frequent	373 (62.9)	220 (37.1)	
Very common	221 (70.8)	91 (29.2)	
**Failed course during school stage**			**<0.001**
No	469 (65.7)	245 (34.3)	
Yes	335 (56.0)	264 (44.1)	
**In love**			**0.005**
No	325 (66.1)	167 (33.9)	
Yes	479 (58.3)	342 (41.7)	
**Alcohol consumption**			**0.001**
Never	658 (64.1)	369 (35.9)	
Monthly or less	83 (50.6)	81 (49.4)	
2 to 4 times a month	42 (50.6)	41 (49.4)	
2 to 3 times a week	16 (64.0)	9 (36.0)	
4 or more times a week	5 (35.7)	9 (64.3)	
**Cigarette smoking**			**0.056**
Never	764 (62.0)	469 (38.0)	
<10 cigarettes/day	17 (40.5)	25 (59.5)	
11 to 20 cigarettes/day	15 (65.2)	8 (34.8)	
21 to 30 cigarettes/day	4 (66.7)	2 (33.3)	
31 or more cigarettes/day	4 (44.4)	5 (55.6)	
**Seek mental health support**			**<0.001**
No	678 (65.3)	360 (34.7)	
Yes	126 (45.8)	149 (54.2)	
**Frequency of social media use during the COVID-19 pandemic**			**<0.001**
Never	89 (77.4)	26 (22.6)	
A bit	189 (66.6)	99 (34.4)	
Moderate	242 (66.7)	121 (33.3)	
A lot	244 (56.9)	185 (43.1)	
Extreme	40 (33.9)	78 (66.1)	
**Frequency of daily internet use**			**<0.001**
1 to 5	532 (65.7)	278 (34.3)	
6 to 10	153 (51.2)	146 (48.8)	
11 to 15	119 (58.3)	85 (41.7)	
**Frequency of daily television use**			0.561
1 to 5	746 (61.5)	467 (38.5)	
6 to 10	43 (60.6)	28 (39.4)	
11 to 15	15 (51.7)	14 (48.3)	
**Family member died from COVID-19**			**<0.001**
No	481 (65.5)	253 (34.5)	
Yes	323 (55.8)	256 (44.2)	
**Acne on face**			**<0.001**
No	433 (64.7)	236 (35.3)	
Grade 1	336 (60.1)	223 (39.9)	
Grade 2	25 (41.7)	35 (58.3)	
Grade 3	1 (14.3)	6 (85.7)	
Grade 4	9 (50.0)	9 (50.0)	
**Family dysfunction**			**<0.001**
No	337 (68.4)	156 (31.6)	
Mild	138 (57.3)	103 (42.7)	
Moderate	52 (42.6)	70 (57.4)	
Severe	217 (59.8)	146 (40.2)	
**Resilience**			0.150
Low	650 (60.2)	430 (39.8)	
High	147 (65.3)	78 (34.7)	
**Victim of Bullying**			0.896
No	520 (61.1)	331 (38.9)	
Yes	284 (61.5)	178 (38.5)	
**Quality of life due to acne**			**0.003**
It doesn’t affect anything-small	660 (62.7)	393 (37.3)	
Moderate-extreme effect	102 (51.5)	96 (48.5)	
**Eating disorder**			**<0.001**
No	548 (72.9)	204 (27.1)	
Yes	205 (42.3)	280 (57.7)	
**Self-esteem**			**<0.001**
High/Medium	500 (68.9)	226 (31.1)	
Low	304 (51.8)	283 (48.2)	

* *p*-value calculated with the Chi Square Test of Independence; bold values indicate statistically significant differences (*p* < 0.05).

**Table 3:** Factors associated with insomnia in schoolchildren from five schools in northern Peru, in simple and multiple regression analysis.

Characteristics	Insomnia					
	Simple Regression			Multiple Regression *		
	PR	IC 95%	*p* **	PR	IC 95%	*p* **
**Categorized age (years)**						
Early	Ref.					
Average	1.04	0.85–1.28	0.692			
Late	0.93	0.81–1.08	0.346			
**Sex**						
Female	Ref.			Ref.		
Male	0.73	0.67–0.80	**<0.001**	0.92	0.78–1.10	0.374
**Type of institution**						
National	0.99	0.87–1.12	0.836			
Particular						
**Place of residence**						
Rural	Ref.			Ref.		
Urban	1.13	0.99–1.27	**0.044**	1.22	1.08–1.38	**0.001**
Marginal urban	0.89	0.52–1.53	0.683	1.13	0.63–2.02	0.679
**Number of members in your family (categorized)**						
1 to 5	Ref.					
6 to 10	1.05	0.95–1.16	0.359			
11 to 15	1.13	0.85–1.50	0.388			
**Religion**						
Any	Ref.			Ref.		
Catholic	1.21	1.05–1.40	**0.009**	1.20	0.93–1.54	0.162
Not Catholic	1.19	1.04–1.36	**0.012**	1.20	1.01–1.41	**0.040**
**Family mental history**						
No	Ref.			Ref.		
Yes	1.50	1.25–1.81	**<0.001**	1.07	0.90–1.26	0.436
**Categorized BMI**						
Underweight	Ref.					
Normal	0.96	0.90–1.02	0.144			
Overweight	1.03	0.91–1.17	0.653			
Obesity	0.98	0.78–1.25	0.897			
**Getting closer to friends**						
Infrequent	Ref.					
Frequent	0.91	0.80–1.04	0.156			
Very common	1.01	0.92–1.12	0.773			
**Approach with relatives**						
Infrequent	Ref.			Ref.		
Frequent	0.77	0.70–0.84	**<0.001**	0.96	0.79–1.17	0.686
Very common	0.60	0.40–0.90	**0.013**	0.78	0.56–1.08	0.137
**Failed course during school stage**						
No	Ref.			Ref.		
Yes	1.28	1.07–1.55	**0.009**	1.13	0.97–1.31	0.115
**In love**						
No	Ref.			Ref.		
Yes	1.23	1.11–1.38	**<0.001**	1.04	0.99–1.09	0.164
**Alcohol consumption**						
Never	Ref.			Ref.		
Monthly or less	1.38	1.17–1.62	**<0.001**	1.06	0.84–1.34	0.633
2 to 4 times a month	1.38	1.01–1.88	**0.042**	0.92	0.70–1.22	0.575
2 to 3 times a week	1.00	0.30–3.40	0.994	0.80	0.30–2.17	0.666
4 or more times a week	1.79	0.85–3.78	0.125	0.96	0.25–3.65	0.952
**Cigarette smoking**						
Never	Ref.			Ref.		
<10 cigarettes/day	1.57	1.29–1.91	**<0.001**	1.20	0.95–1.52	0.127
11 to 20 cigarettes/day	0.92	0.57–1.47	0.716	0.78	0.57–1.05	0.105
21 to 30 cigarettes/day	0.88	0.35–2.22	0.783	0.84	0.52–1.37	0.493
31 or more cigarettes/day	1.46	1.15–1.86	**0.002**	0.90	0.53–1.52	0.693
**Seek mental health support**						
No	Ref.			Ref.		
Yes	1.57	1.42–1.73	**<0.001**	1.18	1.05–1.34	**0.007**
**Frequency of social media use during the COVID-19 pandemic**						
Never	Ref.			Ref.		
A bit	1.49	0.98–2.26	0.062	1.41	0.85–2.34	0.182
Moderate	1.46	0.78–2.73	0.233	1.39	0.66–2.95	0.384
A lot	1.89	1.19–3.02	**0.008**	1.62	0.91–2.90	0.102
Extreme	2.89	1.68–4.99	**<0.001**	2.11	1.11–3.92	**0.018**
**Frequency of daily internet use**						
1 to 5	Ref.			Ref.		
6 to 10	1.43	1.24–1.64	**<0.001**	1.22	1.07–1.40	**0.003**
11 to 15	1.22	1.02–1.46	**0.032**	1.04	0.87–1.24	0.675
**Frequency of daily television use**						
1 to 5	Ref.					
6 to 10	1.03	0.79–1.33	0.846			
11 to 15	1.26	0.99–1.60	0.064			
**Family member died from COVID-19**						
No	Ref.			Ref.		
Yes	1.29	1.04–1.59	**0.019**	1.16	1.01–1.34	**0.035**
**Acne on face**						
No	Ref.			Ref.		
Grade 1	1.14	0.97–1.34	0.115	1.09	0.93–1.28	0.266
Grade 2	1.66	1.37–2.02	**<0.001**	1.41	1.22–1.64	**<0.001**
Grade 3	2.44	2.05–2.91	**<0.001**	1.77	1.22–2.57	**0.002**
Grade 4	1.43	1.25–1.62	**<0.001**	1.19	0.86–1.65	0.294
**Family dysfunction**						
No	Ref.			Ref.		
Mild	1.35	1.12–1.63	**0.002**	1.23	1.05–1.45	**0.012**
Moderate	1.80	1.33–2.42	**<0.001**	1.38	1.06–1.81	**0.017**
Severe	1.26	0.96–1.67	0.098	1.17	0.95–1.44	0.138
**Resilience**						
Low	Ref.					
High	0.87	0.72–1.05	0.154			
**Victim of Bullying**	Ref.					
No	0.98	0.84–1.15	0.824			
Yes						
**Quality of life due to acne**						
It doesn’t affect anything-small	Ref.			Ref.		
Moderate-extreme effect	1.30	1.05–1.62	**0.016**	0.99	0.79–1.24	0.938
**Eating disorder**						
No	Ref.			Ref.		
Yes	2.12	1.82–2.46	**<0.001**	1.72	1.45–2.04	**<0.001**
**Self-esteem**						
High/Medium	Ref.			Ref.		
Low	1.55	1.31–1.82	**<0.001**	1.24	1.13–1.36	**<0.001**

* Adjusted for covariates of interest; ** *p*-values obtained with Generalized Linear Models (GLM), Poisson family, log link function, robust variance, school as cluster; bold values indicate statistically significant differences (*p* < 0.05).

## Data Availability

The datasets generated and analyzed during this study are not publicly available due to participant confidentiality but are available from the corresponding author upon reasonable request.

## Abbreviations

The following abbreviations are used in this manuscript:

BMI	Body Mass Index
CD-RISC	Connor–Davidson Resilience Scale (abbreviated version)
CI	Confidence Interval
CIs	Confidence Intervals
COVID-19	Coronavirus Disease 2019
DLQI	Dermatology Life Quality Index
EBIPQ	European Bullying Intervention Project Questionnaire
ED	Eating Disorder
GLM	Generalized Linear Model
IRR	Incidence Rate Ratio
ISI	Insomnia Severity Index
OR	Odds Ratio
PR	Prevalence Ratio
SCOFF	Sick, Control, One stone, Fat, Food (screening questionnaire for eating disorders)
